# Supplementary material for: Tryptophanyl-Transfer RNA Synthetase Is Involved in a Negative Feedback Loop Mitigating Interferon-γ-Induced Gene Expression
Source: Cells. 2024 Jan 17;13(2):180. doi: 10.3390/cells13020180 (PMC10813977; doi:10.3390/cells13020180)
Supplement: Supplementary file 1 [file cells-13-00180-s001.zip › Supplementary Figures.pdf]

Supplementary Figure S1

Proteins upregulated (76) in A375 cells after 48h of IFN-γ

Biological Process (Gene Ontology)

GO-term	description	count in network	strength	false discovery rate
GO:0002376	Immune system process	47 of 2121	0.76	1.06e-22
GO:0019882	Antigen processing and presentation	18 of 93	1.71	4.18e-21
GO:0006955	Immune response	38 of 1321	0.88	5.91e-21
GO:0044419	Biological process involved in interspecies interaction betw...	38 of 1490	0.83	2.90e-19
GO:0048002	Antigen processing and presentation of peptide antigen	15 of 60	1.82	6.54e-19
GO:0051707	Response to other organism	36 of 1328	0.85	6.54e-19
GO:0006952	Defense response	36 of 1394	0.83	2.01e-18
GO:0098542	Defense response to other organism	30 of 989	0.9	2.01e-16
GO:0045087	Innate immune response	27 of 754	0.97	3.57e-16
GO:0019883	Antigen processing and presentation of endogenous antigen	11 of 25	2.06	7.91e-16
GO:0002483	Antigen processing and presentation of endogenous peptid...	10 of 18	2.16	4.84e-15
GO:0019884	Antigen processing and presentation of exogenous antigen	12 of 48	1.82	4.84e-15
GO:0050776	Regulation of immune response	27 of 844	0.92	4.84e-15
GO:0002478	Antigen processing and presentation of exogenous peptide ...	11 of 39	1.87	3.54e-14
GO:0002682	Regulation of immune system process	32 of 1438	0.77	3.54e-14
GO:0009605	Response to external stimulus	39 of 2355	0.64	4.38e-14
GO:0002831	Regulation of response to biotic stimulus	19 of 361	1.14	1.39e-13
GO:0019885	Antigen processing and presentation of endogenous peptid...	8 of 12	2.24	2.78e-12
GO:0002474	Antigen processing and presentation of peptide antigen via ...	9 of 28	1.93	8.44e-12
GO:0034097	Response to cytokine	23 of 804	0.88	1.56e-11
GO:0031347	Regulation of defense response	21 of 638	0.94	1.85e-11
GO:0045088	Regulation of innate immune response	15 of 238	1.22	2.00e-11
GO:0034341	Response to interferon-gamma	12 of 127	1.39	1.01e-10

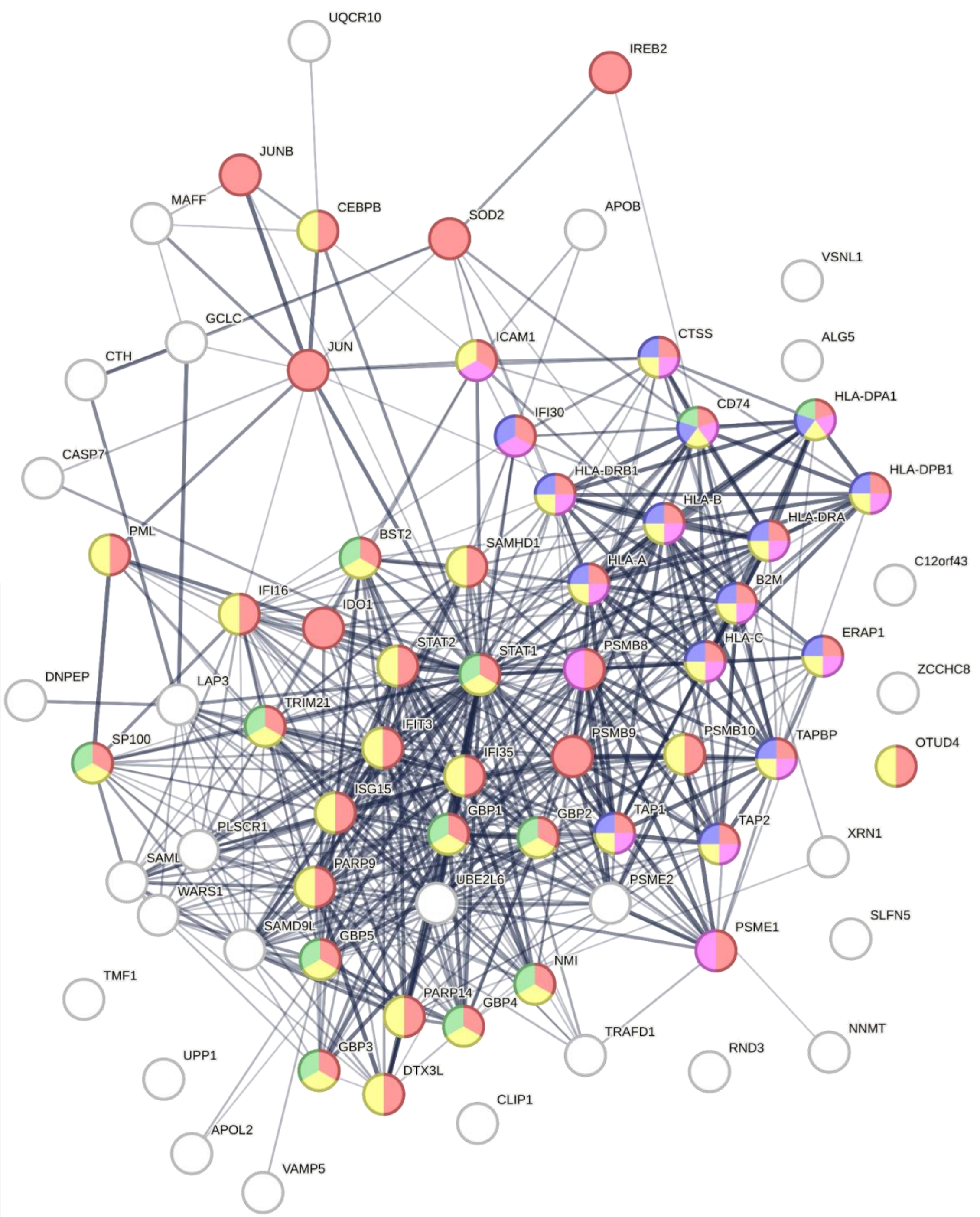

Supplementary Figure S2

Proteins downregulated (184) in A375 cells after 48h of IFN-γ

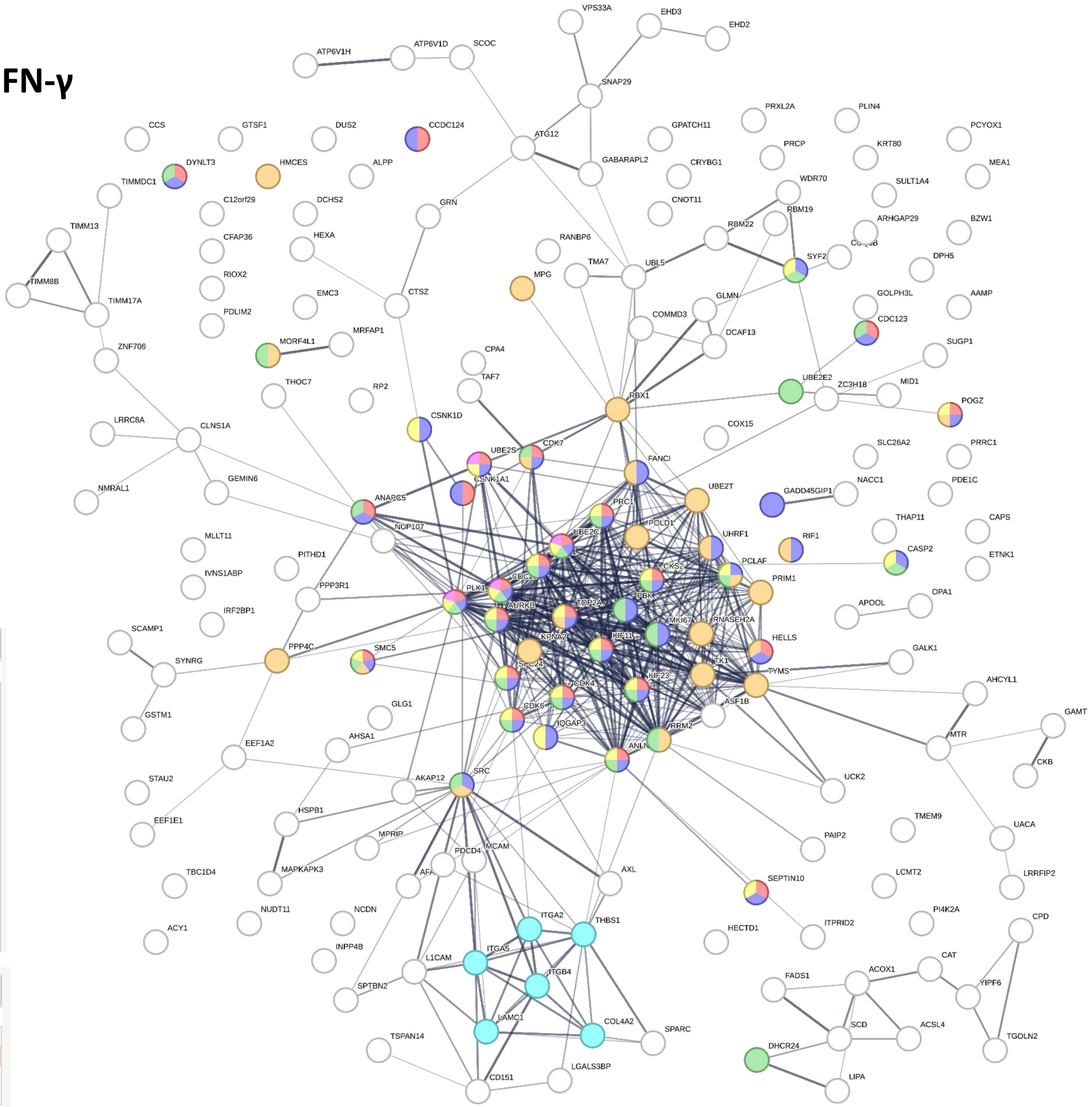

GO-term	description	count in network	strength	false discovery rate
GO:0051301	Cell division	25 of 527	0.69	1.60e-06
GO:0007049	Cell cycle	37 of 1246	0.49	8.46e-06
GO:0044237	Cellular metabolic process	96 of 6568	0.18	0.0033
GO:0007346	Regulation of mitotic cell cycle	18 of 493	0.58	0.0062
GO:1903047	Mitotic cell cycle process	19 of 537	0.57	0.0062
GO:0006259	DNA metabolic process	23 of 785	0.48	0.0071
GO:0000278	Mitotic cell cycle	20 of 631	0.52	0.0077
GO:0051726	Regulation of cell cycle	28 of 1108	0.42	0.0077
GO:0008152	Metabolic process	108 of 7988	0.15	0.0077
GO:0051983	Regulation of chromosome segregation	9 of 128	0.87	0.0094
GO:1901990	Regulation of mitotic cell cycle phase transition	14 of 332	0.64	0.0094
GO:0022402	Cell cycle process	23 of 835	0.46	0.0096
GO:0071704	Organic substance metabolic process	102 of 7522	0.15	0.0103
GO:1904668	Positive regulation of ubiquitin protein ligase activity	4 of 11	1.58	0.0111

Biological Process (Gene Ontology)

pathway	description	count in network	strength	false discovery rate
hsa04110	Cell cycle	8 of 120	0.84	0.0104
hsa04512	ECM-receptor interaction	6 of 88	0.85	0.0465
hsa05222	Small cell lung cancer	6 of 92	0.83	0.0465
hsa04120	Ubiquitin mediated proteolysis	7 of 134	0.74	0.0465

KEGG Pathways

Supplementary Figure S3

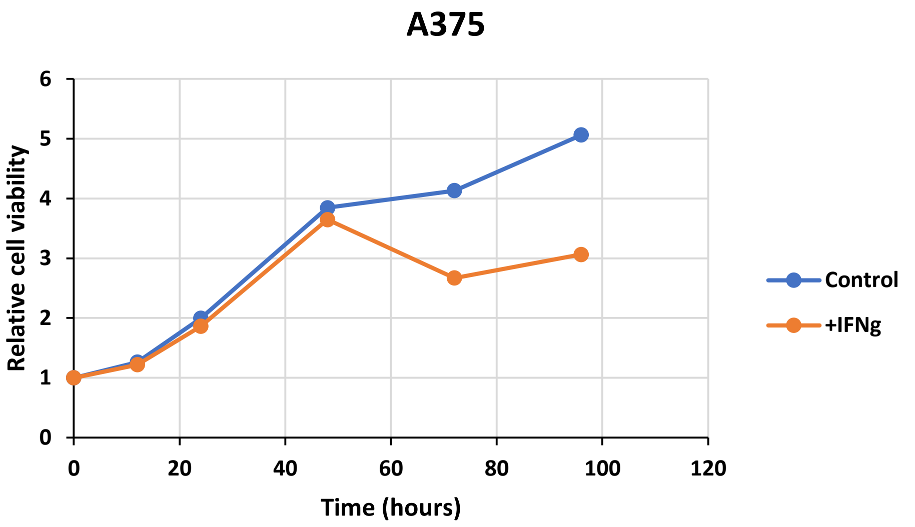

Supplementary Figure S4

TCGA and Gtex data (Tumors and normal tissues)

Comparison of the expression profile of WARS and all the genes from TCGA and Gtex (cuff-off = Top 50 correlating genes)

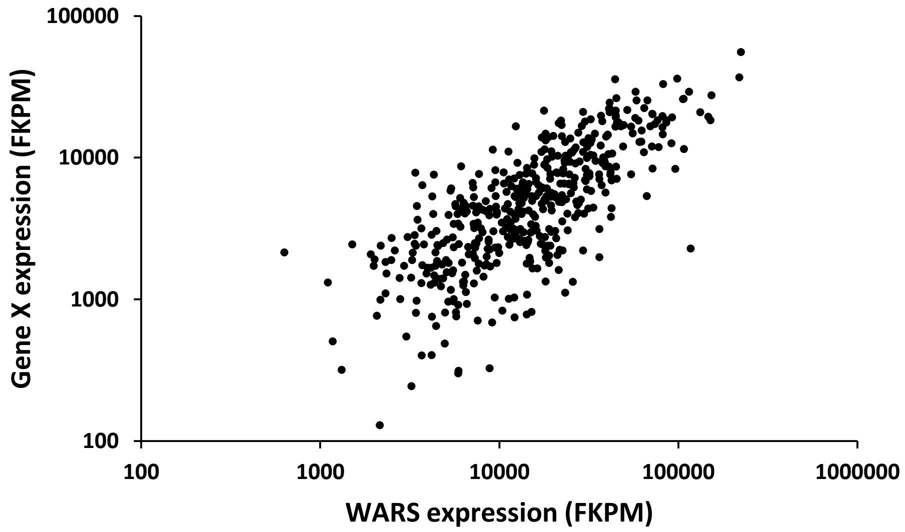

Top 50 correlating genes correlating with WARS

Gene Symbol	Gene ID	PCC
TAP1	ENSG00000168394.10	0.65
STAT1	ENSG00000115415.18	0.59
TAP2	ENSG00000204267.13	0.58
GBP5	ENSG00000154451.14	0.58
BATF2	ENSG00000168062.9	0.56
LAP3	ENSG00000002549.12	0.53
GBP1	ENSG00000117228.9	0.51
CXCL11	ENSG00000169248.12	0.49
GBP4	ENSG00000162654.8	0.49
GBP1P1	ENSG00000225492.6	0.48
PSMB9	ENSG00000240065.7	0.48
UBE2L6	ENSG00000156587.15	0.48
ETV7	ENSG00000010030.13	0.47
CXCL9	ENSG00000138755.5	0.47
CXCL10	ENSG00000169245.5	0.46
HLA-A	ENSG00000206503.11	0.45
LAG3	ENSG00000089692.8	0.45
EPSTI1	ENSG00000133106.14	0.45
APOL6	ENSG00000221963.5	0.44
PSMB8	ENSG00000204264.8	0.44
IL18BP	ENSG00000137496.17	0.43
IFI35	ENSG00000068079.7	0.43
TRIM21	ENSG00000132109.9	0.43

Supplementary Figure S5

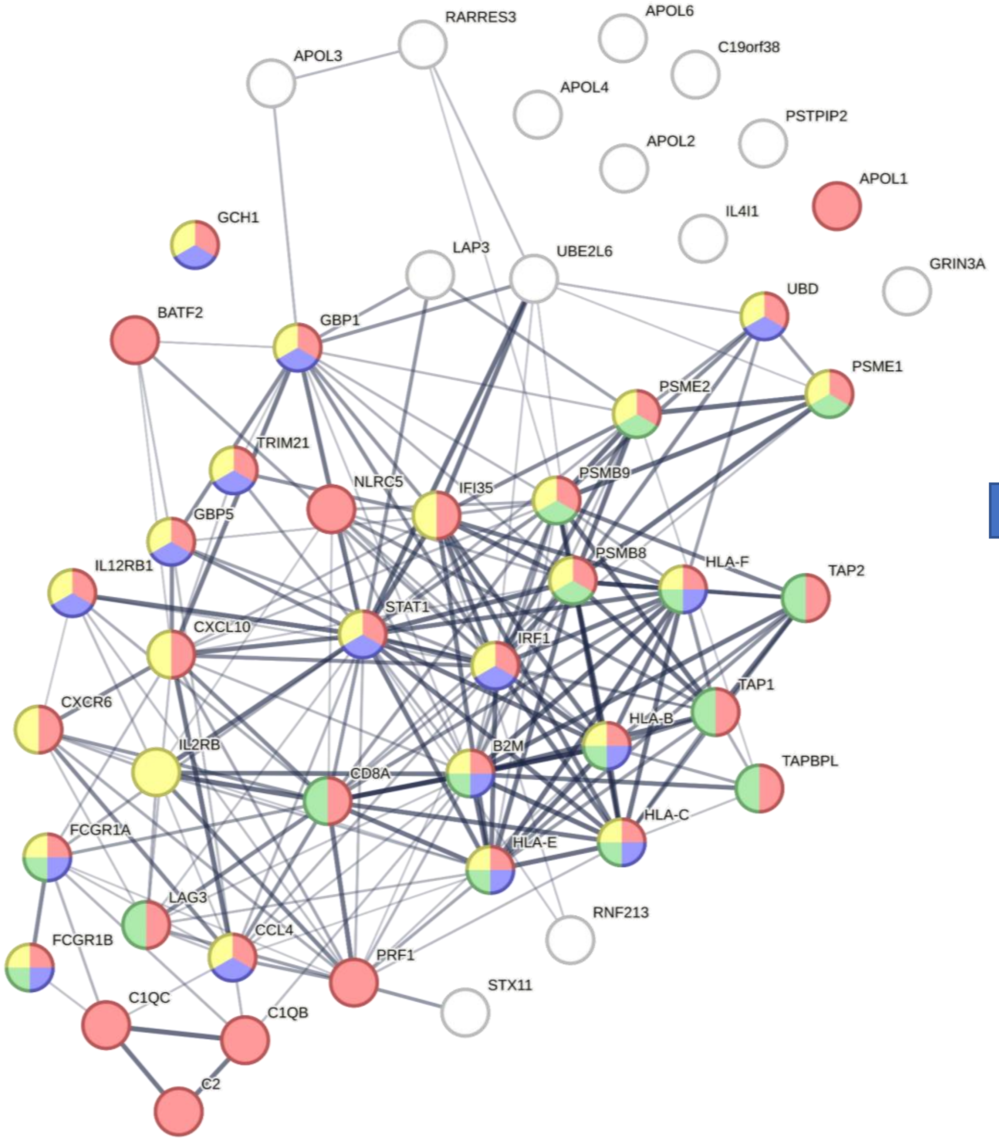

Patient survival

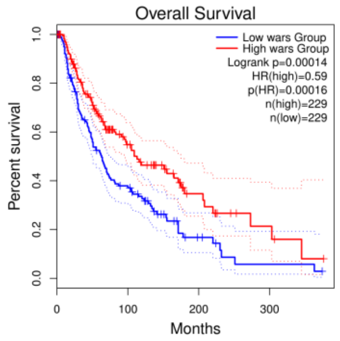

Study Name	Study Abbreviation
Adenoid cystic carcinoma	ACC
Bladder Carcinoma	BLCA
Breast cancer	BRCA
Cervical Squamous Cell Carcinoma and Endocervical Adenocarcinoma	CESC
Cholangiocarcinoma	CHOL
Colon Adenocarcinoma	COAD
Lymphoid Neoplasm Diffuse Large B-cell Lymphoma	DLBC
Esophageal Carcinoma	ESCA
Glioblastoma multiforme	GBM
Head-Neck Squamous Cell Carcinoma	HNSC
Kidney Chromophobe	KICH
Kidney Renal Clear Cell Carcinoma	KIRC
Cervical Kidney renal papillary cell carcinoma	KIRP
Acute Myeloid Leukemia	LAML
Low Grade Glioma	LGG
Liver Hepatocellular Carcinoma	LIHC
Lung Adenocarcinoma	LUAD
Lung Squamous Cell Carcinoma	LUSC
Mesothelioma	MESO
Ovarian Cancer	OV
Pancreatic Ductal Adenocarcinoma	PAAD
Pheochromocytoma and Paraganglioma	PCPG
Prostate adenocarcinoma	PRAD
Rectum Adenocarcinoma	READ
Sarcoma	SARC
Skin cutaneous melanoma	SKCM
Stomach Adenocarcinoma	STAD
Testicular Germ Cell Tumors	TGCT
Thyroid carcinoma	THCA
Thymoma	THYM
Uterine Corpus Endometrial Carcinoma	UCEC
Uterine Carcinosarcoma	UCS
Uveal Melanoma	UVM

Supplementary Figure S6

A

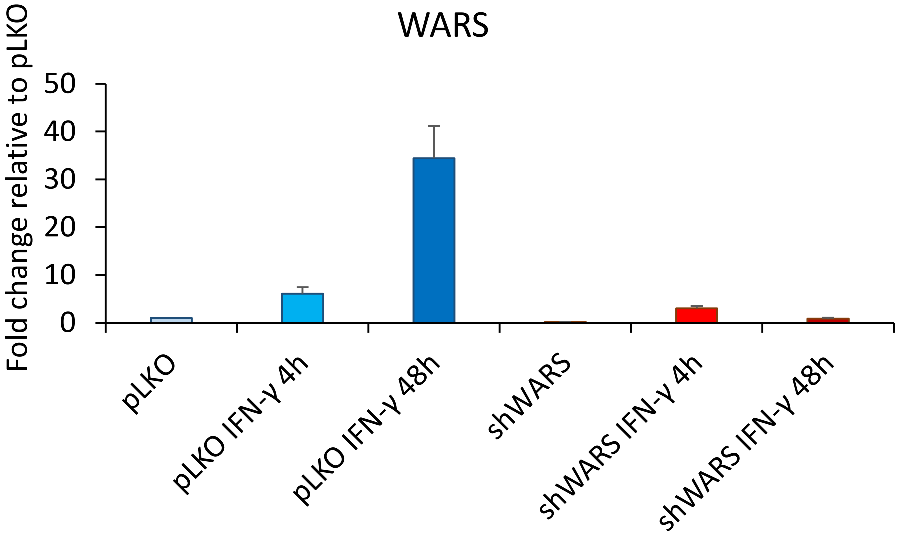

B

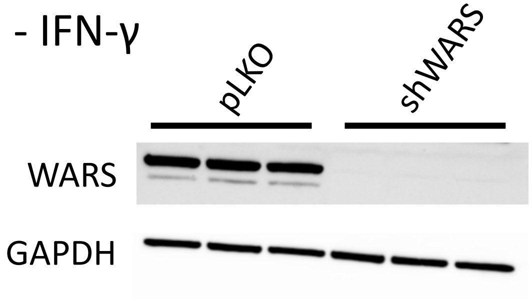

C

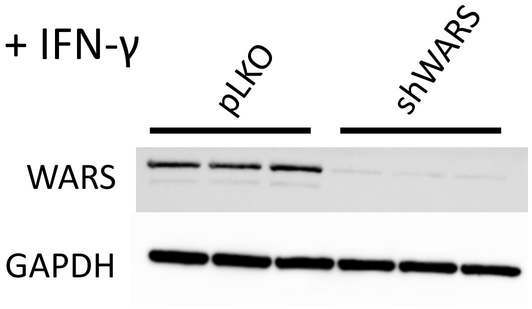

Supplementary Figure S1: Proteins up-regulated in A375 cells following interferon-γ treatment and enriched pathways.

Supplementary Figure S2: Proteins down-regulated in A375 cells following interferon-γ treatment and enriched pathways.

Supplementary Figure S3: Cell viability assay of A375 cells treated with vehicle or interferon-γ. 5,000 A375 cells were seeded in white 96-well plates and treated with 100ng/mL of IFN-γ. Cell viability was monitored every 24h for 4 days using Cell Titer Glo kit (Promega).

Supplementary Figure S4: Identification of genes correlating with *WARS* using GEPIA.

Supplementary Figure S5: Cancer patients survival analysis of genes correlating with *WARS* using GEPIA.

Supplementary Figure S6: Inhibition of WARS expression by shRNA at the RNA (A) and protein (B-C) levels in A375 cells treated with vehicle (-IFN-γ) or interferon-γ (+IFN-γ).
